# The Role of Training Age and Sport Specialization in Achieved Selection Tier in Adolescent Handball Talents

**DOI:** 10.1186/s40798-026-01079-w

**Published:** 2026-08-11

**Authors:** Lutz Thieschäfer, Jörg Schorer, Urs Granacher, Dirk Büsch

**Affiliations:** 1 Sport and Training Department, Institute of Sport Science, Carl von Ossietzky Universität Oldenburg, Oldenburg, Germany; 2 Sport and Movement Science Department, Institute of Sport Science, Carl von Ossietzky Universität Oldenburg, Oldenburg, Germany; 3 Department of Sport and Sport Science, Exercise and Human Movement Science, University of Freiburg, Freiburg, Germany

**Keywords:** Diversification, Secondary data, Talent selection, Unisport vs. multisport, Youth

## Abstract

**Background:**

The debate over whether beginning a sport early and specializing in it promotes athletic success in adulthood has been the subject of academic discussion for decades. Current evidence suggests the opposite. Multisport participation during youth and a later start in the primary sport may positively influence athletic success in adulthood, although the effects on youth athletes remain inconclusive. However, youth sports performance and developmental potential are central to success in talent selection processes, which decisively shape athletes’ subsequent talent development. Therefore, this study aimed to determine the handball training age and prevalence rates of sport specialization in talented female U15 and male U16 handball players and to examine their associations with the attained selection tier. In addition, the years of practice in other sports (i.e., depth), the number of other sports (i.e., breadth), and the type of other sports were compared between selection tiers.

**Results:**

The vast majority of female (92.8%) and male (97.0%) players were classified as specialized athletes. Almost one third of the female (30.9%) and male players (30.8%) have never been involved in sports other than handball (i.e., single-sport players). Multisport engagement was not significantly associated with the selection tier in both sexes. Furthermore, there were no adverse associations between the breadth, depth, and type of other sports practiced and the selection tier. Female (*g* = 0.59) and male (*g* = 0.40) players who achieved higher selection tiers demonstrated a higher training age compared to non-selected players.

**Conclusions:**

In light of the players’ mid-teen age, the high prevalence rates of sport specialization were as expected. Nevertheless, the relatively high proportion of single-sport players may give cause for concern, particularly given that multisport engagement did not impede players from reaching high selection tiers. In fact, multisport engagement can be considered a viable alternative developmental pathway toward adult high performance. Furthermore, training age was associated with selection tier. Consequently, having a high training age may increase the likelihood of being selected as a “talent” which should be considered in talent selection processes.

## Key Points

- A substantial proportion of talented female U15 and male U16 handball players can be classified as sport specialized, with nearly one third identified as single-sport athletes.
- Multisport engagement was not detrimental to achieving a high selection tier and can therefore be considered a viable alternative to the sport specialization pathway.
- Training age was positively associated with selection tier, suggesting that a higher training age may increase the likelihood of being identified as a “talent”, which has important implications for talent selection processes.

## Background

A common belief among many coaches, parents, and athletes is that early and exclusive dedication to a single sport (i.e., sport specialization) is conducive to attaining superior athletic performance and greater career success [1–4]. This notion is presumably driven by the contention that early and high amounts of deliberate practice in one sport are directly linked to the development of expertise and achievement within that sport [5]. Focusing on a single sport from an early age (i.e., a high training age) facilitates the accumulation of time spent on deliberate practice and is thus considered a logical pathway toward adult high performance [6]. Although the concept of “early sports specialization” is extensively discussed in the literature, it is important to acknowledge that sport specialization and commencing a sport at an early age are not inherently concomitant. For instance, athletes may enter their primary sport at an early age and concurrently engage in other sports. Conversely, they may start their primary sport late and subsequently devote themselves exclusively to that sport, indicating potential alternative pathways toward adult excellence.

Sport specialization and multisport engagement are antagonistic concepts, both aiming at developing young and promising athletes toward high performance athletes [7, 8]. Regarding sport specialization, we refer to the consensus definition of “intentional and focused participation in a single sport for a majority of the year that restricts opportunities for engagement in other sports and activities” [9]. In addition, athletes may also be considered specialized if they have only ever been involved in one sport (i.e., single-sport athletes). In comparison, multisport engagement has previously been defined as “involvement in a number of different sports before specializing in later stages of development” [10]. It is characterized by practice (i.e., diversification) and play (i.e., sampling) in several sports during childhood (i.e., sampling years) instead of focusing on a single sport [11]. A certain degree of sport specialization is inevitable to reach elite status [7], but this occurs later after the sampling years, when involvement in other sports subsequently declines in the early teen years (i.e., specializing years) and eventually ceases in the late teen years (i.e., investment years) [11, 12]. However, the time at which specialization occurs depends on the type of sport and the age at which peak performance is typically reached in that sport [13]. The rationale behind the multisport pathway toward high performance is that practice and play in a breadth of sports hypothetically cross-pollinate skill and physical development in the main sport by developing transferable general capabilities [6, 14]. Identical elements, such as movement, perceptual, conceptual, and conditioning elements, that sports have in common may positively transfer between these sports when appropriately developed, thus leading to performance improvements [10].

The question of which path to long-term sporting success in adulthood outperforms the other path is open for debate. A meta-analysis by Macnamara et al. [15] suggests that deliberate practice alone does not fully explain later sporting success in adulthood. These authors demonstrated that merely 18% of the variance in athletes’ later level of sport performance can be explained by differences in the amount of deliberate practice performed. When examining the subpopulation of top international athletes only, the proportion of explained variance is reduced to just 1%. In addition, high-performing athletes did not begin their sport earlier in childhood than lower-performing athletes, suggesting that training age alone may be a less critical factor contributing to sports performance in adulthood. In terms of specialization, it is widely accepted that participating in multiple sports at an early age will not deter athletes from achieving long-term success in sports, where peak performance is typically attained in adulthood [3, 16–19]. In fact, a plethora of retrospective studies have demonstrated that highly successful adult athletes (e.g., medalists and world-class athletes) had a later main-sport start and greater amounts of (coach-led) multisport practice during childhood than their lower-performing controls [20–25]. Furthermore, adult athletes with multisport background participated in more games, suffered from fewer major injuries, and had longer careers [24, 26]. The multisport pathway appears to not only increase the likelihood of becoming a successful high performance adult athlete, but may also provide certain advantages for youth athletes. There is a large body of evidence showing that (early) sport specialization increases the risk of sustaining (overuse) injuries in young athletes [24, 27–32]. In addition, prevalence rates for suffering from fatigue, anxiety, depression, and burnout as well as associations with trauma-related disorders are higher in specialized versus non-specialized athletes [33–35]. All of these are factors that may prompt young athletes to drop out of their sport [6, 32, 33]. In summary, multisport engagement and a later entry into the main sport appear to provide certain advantages in different aspects for both youth and adult athletes [3].

Discussion about the appropriate athletic pathway may fall short when only examining the final outcome; that is whether a talented young athlete becomes a high performance athlete or not. In fact, talent selection or deselection during talent development processes may also impact on the likelihood of becoming a high performance athlete. For many sports, the early teenage years are a crucial period for entry into national talent development programs (TDP), which can pave the way for later success [36]. The selection of athletes as “talents” and their subsequent enrollment in talent development programs or nomination for national squads is typically contingent upon their successful navigation of a series of talent selection tiers. These talent selection processes incrementally demand high athletic and sport-specific performance, in addition to substantial developmental potential. Consequently, the potential repercussions of sports specialization/multisport engagement and training age on junior-age sports performance could also have an impact on the achieved selection tier and entry into talent development systems (TDS) and are therefore a worthwhile subject of study.

Although the positive long-term outcomes of the multisport pathway are evident, the early consequences of specialization/multisport engagement for youth athletes regarding performance outcomes and sporting success are inconclusive. In their systematic reviews, Kliethermes et al. [16] and Luo et al. [28] reported either an absence or a negative association between sport specialization and performance outcomes, such as physical fitness or coordination. The results in youth athletes suggest that the positive effects of sport sampling may initially be latent and emerge later on during the athletic career [3, 37]. Furthermore, research has demonstrated that the range and extent of involvement in other sports are either insignificantly or even positively associated with sports success at the youth level [14, 38–40]. For instance, Baker et al. [14] observed that (during handball talent selection camps) selected female players participated in a greater number of sports and accumulated more hours of training in other sports than their non-selected peers, although this was not evident among male players. On the other side, meta-analyses revealed contrasting results, indicating that the predictors of senior elite success were antithetical to the predictors of junior-age success [20–22]. Following this, the attainment of junior-age athletic success was found to be facilitated by three factors that correspond to the deliberate practice approach [5]: an earlier start in the main sport, more practice in that sport, and less practice in other-sports. Overall, there is ambiguity in the contemporary body of evidence on the association between multisport commitment and sporting success in youth athletes. With regard to training age, entering the main sport earlier may be conducive to attaining higher performance at the youth level.

Given the differences in starting age, age of specialization, and TDS between sports, there has been a recent call for additional empirical research at a sport-specific level to enhance the existing knowledge base on talent development processes [13]. Therefore, this paper focuses on handball, a field with limited evidence on the appropriate pathway. Early engagement in handball combined with cross-over sampling of various sports during childhood and youth are considered key training and development features [41]. Male handball players commence their sport on average at around 9.1 years of age and specialize approximately at age 10.0 [42]. Whereas, in high performance handball, female players typically begin playing handball at approximately 9.0 years of age and start specializing at age 14.0, while male players start playing at approximately 9.6 years of age and begin specializing at age 13.4 [13]. These early teenage years of specialization are a pivotal junction for young handball players in which the course for further talent development is set. Key entry points (e.g., scouting camps, tournaments) into handball TDS usually occur during the transition between players’ specializing years and investment years [11, 43, 44]. Recent findings have indicated that later sporting success in high performance handball is not merely a matter of chance but rather the consequence of effective talent development [36]. It is therefore worthwhile to achieve a high selection tier to successfully making the leap into TDP that will increase the likelihood of becoming a high performance level handball player.

Accordingly, the aims of this study were threefold: First (1), to examine the prevalence of sport specialization among youth handball players at the annually conducted National Talent Selection Camp (SelCamp; Ger.: DHB-Sichtung) of the German Handball Federation (DHB; Ger.: Deutscher Handballbund e.V.). With reference to the relevant literature [13], it was hypothesized that a high number of female U15 and male U16 handball players may already be sports specialized. Second (2), to investigate whether a multisport background in general is associated with the achieved selection tier. Given the ambiguous outcomes of previous studies [14, 38–40], it was hypothesized that a multisport background is at least not detrimental to achieving high selection tiers. Additional analyses were conducted on players with multisport background to gain a more comprehensive understanding of the various facets of multisport engagement. Specifically, it was exploratively assessed whether the breadth (i.e., the number of other sports), depth (i.e., the number of years spent in other sports), and type of other sports practiced are associated with the achieved selection tier. Third (3), to examine whether players’ handball training age can differentiate between selection tiers. Consistent with the meta-analyses of Barth et al. [21] and Güllich et al. [22], the hypothesis was that players who achieve a higher selection tier begin playing handball at an earlier age and thus, have a greater training age. Due to potential sex differences in the age of specialization [13] and multisport background [14], the analyses were conducted separately for each sex.

## Methods

We received data from the DHB SelCamps from 2022 to 2025 for female U15 and male U16 handball players separately (Ger.: Landeskader) and squad lists of German youth national teams (Ger.: Nachwuchskader 1/2) from the same period. The dataset encompasses the players’ previous and current involvements in other sports in a club, the specific sports they practiced, and the duration of their involvement as well as their handball training age. Additionally, the players’ selection status as determined by national coaches were provided.

Since this study utilized an ex post facto design with secondary data analysis, it is exempt from ethics approval due to the previously conducted collection and retrospective analysis of anonymized data. Nevertheless, the physical fitness and sport-specific tests were applied by experienced handball coaches and/or strength and conditioning specialists and data acquisition was in accordance with the Declaration of Helsinki including its later amendments. In addition, we adhered to good practice standards for conducting secondary data analyses [45].

### Participants

The dataset included *n* = 879 female U15 players with a mean chronological age (with standard deviations in parentheses) of 14.6 (0.4) years and *n* = 932 male U16 aged 15.6 (0.4) years. The players were divided into three selection tiers: the highest selection tier is represented by the nomination for the youth national team. Players who were part of the youth national team at any point between 2022 and 2025 were designated as “youth national team players” (YNTP), which pertained to *n* = 64 female players and *n* = 65 male players. Players who were appraised as players with potential by the national coaches, but who were not selected in the youth national team were designated as “selected players” (SP), which applied to *n* = 126 female players and *n* = 119 male players. The remaining *n* = 689 female players and *n* = 748 male players who were not further considered for talent development at this point were referred to as “non-selected players” (NSP).

### Procedures

The SelCamp procedure within the DHB’s TDS has been described in detail elsewhere [46]. Briefly, the DHB annually conducts SelCamps to identify talented female U15 and male U16 players for inclusion in development programs and youth national teams [47]. Coaches from 20 regional federations each put forward approximately 12 players per sex to participate in the SelCamps. At the camps, players are assessed using general motor tests, handball-specific performance tests, and evaluations of technical and tactical performance in match situations. Based on these assessments, national coaches select the most promising players for further development. The data collection started shortly before the SelCamps. The players completed an online questionnaire (LimeSurvey GmbH, Hamburg, Germany), which included the items “Have you previously been involved in any sport other than handball in a club?” and “Are you currently involved in any sport other than handball in a club?”. If the participants answered with “Yes”, they were asked to specify the type of sport and the years of involvement in that sport. The questions were repeated until the players answered “No”, allowing them to indicate their involvement in even more sports. Furthermore, the players had to specify how long they had been playing handball in years.

To distinguish current specialization at the time of the SelCamp, players who were at that time not involved in any sport other than handball were categorized as “specialized players”, whereas players who were at that time involved in any sport other than handball were labeled “non-specialized players”. When distinguishing between players’ pathways, players with a multisport background, i.e., previous and/or current involvement in other sports on a club level, were classified as “multisport players”, whereas players who had never been involved in sports other than handball on a club level were considered “single-sport players”.

### Statistical Analysis

Statistical analyses were performed with R (4.5.0) and IBM SPSS Statistics (30.0.0.0, IBM, Armonk, NY, USA). The statistical significance level was established at α = 0.05. The applied statistical procedures were computed separately for female and male players. The numbers in parentheses indicate the respective study aims to be investigated.

1. Prevalence rates for specialized athletes at the time of the SelCamp as well as prevalence rates for single-sport players were reported using frequencies and percentage rates. Wilson Score intervals were calculated and used as 90% confidence interval (CI).
2. To examine associations between players’ current specialization at the time of the SelCamp as well as single-sport/multisport background and selection tier, chi-square tests of independence were employed. Cramér’s *V* and 90% CI were calculated and interpreted with *df* adjustment according to the orientations provided by Cohen [48], with effect size ranges of 0.07 to 0.20, 0.21 to 0.34, and > 0.34, as small, medium, and large effects, respectively. Fisher’s exact test was computed when at least one cell had an expected count below five.
  Additionally, it was exploratively examined whether the breadth, depth, and type of other sports within players with a multisport background are associated with their achieved selection tier: Kruskal–Wallis tests were computed to determine whether the number (i.e., breadth) of other sports practiced can differentiate between selection tiers. One-way analyses of variance (ANOVA) were performed to assess whether the accumulated years (i.e., depth) in other sports differed between the selection tiers of multisport players. Homogeneity of variance was verified using Levene’s test. It should be noted that the sample size was slightly reduced in this analysis due to missing data on the duration of involvement in other sports for *n* = 16 female players and *n* = 6 male players. Cohen’s *d* with 90% CI were calculated. An orientation of small, medium, and large effects was based on Cohen’s *d* ranges of 0.20 to 0.49, 0.50 to 0.79, and > 0.79, respectively [48]. To explore the association between the types of other sports (i.e., invasion sports, track and field, combat sports, and aesthetic sports) practiced by multisport players and their achieved selection tier, chi-square tests were calculated as stated above.
3. One-way ANOVAs were performed as outlined above to examine whether the training age differed between the selection tiers. Additionally, planned contrast analyses were conducted, with weights of − 2, 1, and 1 for NSP, SP, and YNTP selection tiers, respectively, to test the hypothesis that selected players and beyond (i.e., SP and YNTP combined) have a higher handball training age than NSP. Hedges’ *g* with 95% CI were calculated.

In addition, Bayesian statistics were computed to complement the classical frequentist statistics. In the absence of substantial prior knowledge in this field, default priors for fixed effects (*r* scale value = 0.5) were utilized. Interpretations of the Bayes factors were based on the classification provided by Lee and Wagenmakers [49].

## Results

### Prevalence Rates for Sport Specialization

**Fig. 1:**
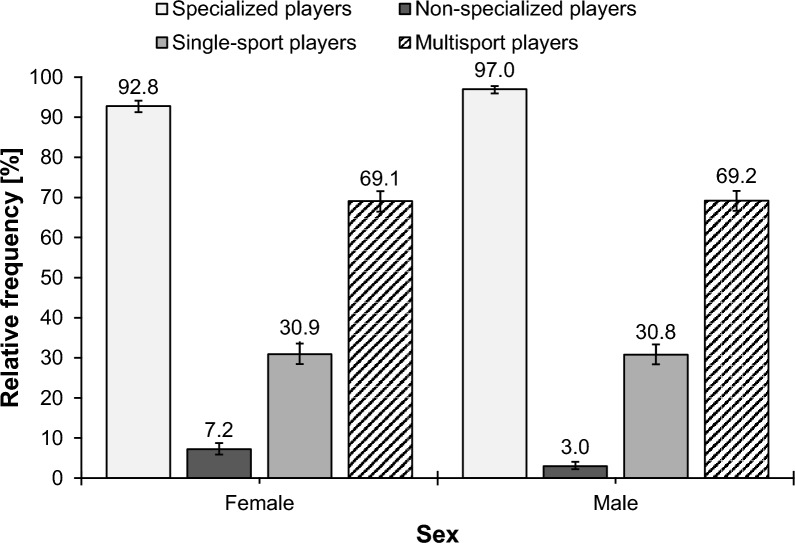
Prevalence of specialization in female and male handball players. Note. Error bars represent the 90% CI. Means are shown above the bars

At the time of the SelCamp, 92.8% (*n* = 816, 90% CI [91.3, 94.1]) of the female players were classified as sport-specialized. Almost one-third (30.9%, *n* = 272, 90% CI [28.4, 33.6]) of the female players had never practiced any other sport at a club level besides handball. Accordingly, these players were classified as single-sport players. The vast majority (97.0%, *n* = 904, 90% CI [95.9, 97.8]) of the male players were sport-specialized at the time of the SelCamp. The proportion of single-sport players among males (30.8%, *n* = 287, 90% CI [28.4, 33.3]) was nearly identical to that reported for female players. Prevalence rates for sport specialization according to sex are illustrated in Fig. 1.

### Associations Between Multisport Engagement and Selection Tier

For female players, sport specialization was not significantly related to the players’ selection tier (*p* = 0.741). The ratios of single-sport and multisport background were similar between NSP, SP, and YNTP, indicating that there was no significant association between single-sport/multisport background and selection tiers (*p* = 0.567). These observations were corroborated by the Bayes factors providing very strong to extreme evidence in favor of the null hypothesis (i.e., no significant association). Analogous outcomes were noted among the male players. Neither sport specialization (*p* = 0.431) nor multisport background (*p* = 0.805) were significantly related to the players’ selection tier, which was underpinned by the Bayes factors indicating very strong to extreme evidence in favor of the null hypothesis. Table 1 shows the results for the association between sport specialization/multisport background and players’ selection tier differentiated by sex.

**Table 1:** Associations between sport specialization and selection tier in female and male players

Sex	Specialization	Selection tier			χ^2^(2)	*p*	*V*	90% CI		1-β	*BF*_*01*_
		NSP	SP	YNTP				*LL*	*UL*		
Female (*n* = 879)	Specialized	640	118	58	0.60	0.741	0.03	< 0.01	0.07	0.10	134.55
	Non-specialized	49	8	6							
	Single-sport background	219	36	17	1.14	0.567	0.04	< 0.01	0.08	0.14	35.89
	Multisport background	470	90	47							
Male (*n* = 932)	Specialized	723	116	65	2.40	0.431^a^	0.05	< 0.01	0.10	0.26	173.77
	Non-specialized	25	3	0							
	Single-sport background	234	34	19	0.43	0.805	0.02	< 0.01	0.06	0.08	49.59
	Multisport background	514	85	46							

^a^Fisher’s exact test

NSP non-selected players, SP selected players, YNTP youth national team players, *V* Cramér’s *V*, CI confidence interval, *LL* lower limit, *UL* upper limit, *BF*_*01*_ Bayes factor

Exploratory analyses were conducted on players with multisport background to gain a more comprehensive understanding of the various aspects of multisport engagement. The number of other sports practiced (i.e., breadth) did not significantly differ between selection tiers in female multisport players (*n* = 607), *H*(2) = 2.34, *p* = 0.310. The median for the number of sports practiced was *Mdn* = 1 in all selection tiers, and the mean ranks were 306.83 for NSP, 281.96 for SP, and 317.89 for YNTP. Similarly, for male multisport players (*n* = 645), the breadth of other sports practiced was not significantly different between selection tiers, *H*(2) = 1.06, *p* = 0.590. The median for the number of sports practiced was *Mdn* = 1 in all selection tiers, and the mean ranks were 325.74 for NSP, 307.95 for SP, and 320.18 for YNTP.

To test whether the depth of involvement in other sports differs across selection tiers, one-way ANOVAs were conducted. The means and standard deviations of accumulated years of practice in other sports for female players were 5.99 (4.18), 6.09 (4.76), and 5.36 (3.03) for NSP, SP, and YNTP, respectively. For male players, the corresponding values were 5.42 (3.58), 4.70 (2.90), 4.98 (3.19) for NSP, SP, and YNTP, respectively. There were no statistically significant differences in accumulated years of practice in other sports between selection tiers. This was the case for female players, *F*(2, 588) = 0.52, *p* = 0.596, *d* = 0.08, 90% CI [< 0.01, 0.19], 1-β = 0.14, *BF*_01_ = 17.40, and for male players, *F*(2, 636) = 1.73, *p* = 0.178, *d* = 0.15, 90% CI [< 0.01, 0.26], 1-β = 0.36, *BF*_01_ = 5.33.

No statistically significant associations were found between the type of other sports practiced and selection tier of female or male players (0.318 ≤ *p* ≤ 0.750), except for aesthetic sports in female players, where a significant association was noted with a small effect size (*p* = 0.040, *V* = 0.10). Compared with their non-selected peers, female YNTP were disproportionately more likely to be involved in aesthetic sports. However, the Bayes factor is close to one and thus provides merely anecdotal evidence, indicating the necessity for additional data. The relations between invasion sports, track and field, combat sports, and aesthetic sports involvement and selection tier are presented in a contingency table (Table 2).

**Table 2:** Associations between type of sport practiced and selection tier in female and male players

Sex	Type of sport	Selection tier			χ^2^(2)	*p*	*V*	90% CI		1-β	*BF*_*01*_
		NSP	SP	YNTP				*LL*	*UL*		
Female (*n* = 607)	Invasion sports	98	14	12	2.12	0.347	0.06	< 0.01	0.12	0.24	19.72
	Other sports	372	76	35							
	Track and field	107	21	13	0.57	0.750	0.03	< 0.01	0.08	0.10	37.54
	Other sports	363	69	34							
	Combat sports	31	8	1	2.29	0.318	0.06	< 0.01	0.12	0.25	53.25
	Other Sports	439	82	46							
	Aesthetic sports	210	40	30	6.43	0.040	0.10	0.02	0.16	–	1.56
	Other sports	260	50	17							
Male (*n* = 645)	Invasion sports	366	65	35	1.37	0.504	0.05	< 0.01	0.10	0.17	24.54
	Other sports	148	20	11							
	Track and field	63	13	8	1.43	0.490	0.05	< 0.01	0.10	0.17	34.53
	Other sports	451	72	38							
	Combat sports	59	7	4	1.03	0.597	0.04	< 0.01	0.09	0.04	64.90
	Other sports	455	78	42							
	Aesthetic sports	36	5	2	0.58	0.750	0.03	< 0.01	0.08	0.10	128.84
	Other sports	478	80	44							

NSP non-selected players, SP selected players, YNTP youth national team players, *V* Cramér’s *V*, CI confidence interval, *LL* lower limit, *UL* upper limit, *BF*_*01*_ Bayes factor

### Associations Between Training Age and Selection Tier

**Fig. 2:**
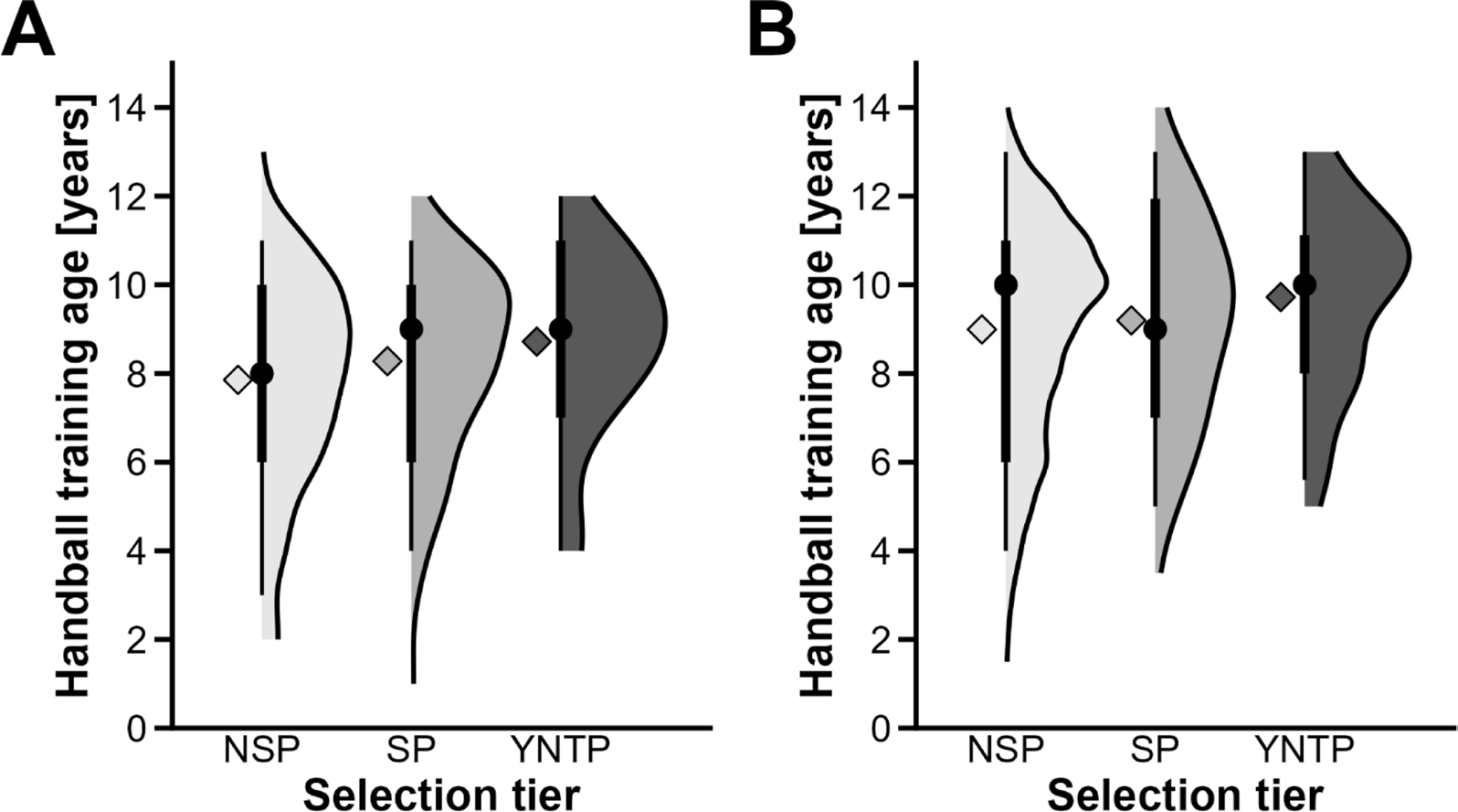
Half-eye plots of handball training age across different selection tiers in female (panel **A**) and male players (panel **B**). The shaded “half-eyes” describe the density of the data distribution, where wider sections indicate higher data density. The medians are represented as dots, while the means are represented as diamonds. The thick and thin vertical bars represent the 66% and 95% quantile intervals, respectively. *NSP* non-selected players; *SP* selected players, *YNTP* youth national team players

The mean handball training age descriptively increased across selection tiers as illustrated in Fig. 2. The means and standard deviations of handball training age for female players were 7.86 (2.23), 8.28 (2.10), and 8.72 (2.01) for NSP, SP, and YNTP, respectively. For male players, the corresponding values were 8.99 (2.38), 9.20 (2.26), 9.72 (2.02) for NSP, SP, and YNTP, respectively. The training age of female YNTP was, on average, 0.86 years greater than that of NSP, and 0.44 years greater compared to SP. Planned contrast analysis revealed a medium effect size in which selected female handball players and above (i.e., SP and YNTP combined) had a higher training age than NSP, *t*(876) = 3.42, *p* < 0.001, *g* = 0.59, 95% CI [0.30, 0.87]. The main effect for selection tier was statistically significant, *F*(2, 876) = 5.89, *p* = 0.003, *d* = 0.23, 90% CI [0.11, 0.33], *BF*_10_ = 6.58. The Bayes factor substantiated this finding with moderate evidence in favor of the main effect model. A comparable difference in training age between YNTP and NSP was observed among males, with a mean difference of 0.73 years. The mean difference in training age between YNTP and SP was 0.52 years. The result of the planned contrast analysis indicated that SP and YNTP combined had a greater training age than NSP, *t*(929) = 2.34, *p* = 0.020, *g* = 0.40, 95% CI [0.12, 0.68]. A statistically significant main effect for selection tier with a small effect size was detected, *F*(2, 929) = 3.11, *p* = 0.045, *d* = 0.16, 90% CI [0.02, 0.26], *BF*_01_ = 2.11. However, it is important to acknowledge that the Bayes factor provides contradictory evidence, as the data were twice (*BF*_01_ = 2.11) as likely under the null model than under the main effect model.

## Discussion

The main findings of this study indicate (1) a high prevalence of current sport specialization in female and male players at the SelCamp, affirming the a priori hypothesis. Furthermore, as hypothesized, (2) neither multisport engagement in general nor its breath, depth, or type of other sport practiced was adversely associated with the achieved selection tier of both female and male players. However, (3) the handball training age was significantly higher in female and male SP and YNTP compared to NSP, thereby corroborating our initial assumptions. These results are explained in more detail in the following sections.

This study was conducted for the purpose of investigating three specific aims: The first aim was (1) to examine the prevalence of sport specialization among players at the SelCamp. At some point in their careers, athletes eventually need to specialize to achieve top-level performance [7]. Although our data did not provide the exact point in time at which players specialized, the vast majority of players were already sport-specialized at the time of the SelCamp, affirming the a priori hypothesis. In high performance handball, the mean specialization ages of female and male players are 14.0 and 13.4 years, respectively [13]. The mean chronological ages of female (14.6 years) and male (15.6 years) players in this sample indicate that the players were on average already beyond the mean specialization age. Consequently, the high prevalence rates of current sport specialization among female (92.8%) and male players (97.0%) observed at the SelCamp are not surprising. This finding is consistent with the results reported by Baker et al. [14], who documented a high degree of specialization among 14- to 16-year-old handball players. Additionally, the mean number of other sports practiced was found to be below one in their sample, indicating the presence of single-sport players. Consequently, the prevalence rates of single-sport players among females (30.9%) and males (30.8%) in the present study appear to be reasonable. It should be noted that specialization does not imply that players are exclusively engaged in handball specific training. The conventional training regimens also encompass, for instance, self-directed strength and endurance training as well [41].

Whether athletes deliberately choose to specialize in a single sport or are driven to do so by sports and educational systems remains unresolved. It is important to acknowledge that the single-sport/specialization pathway is not automatically “incorrect” for every individual athlete [3, 6]. While some athletes are intrinsically motivated to specialize [20, 50], parental and coaching pressures can push early commitment [32, 50], partly due to limited awareness of current recommendations [51]. Consequently, better education and communication among athletes, families, coaches, and organizations are essential to facilitate informed decision-making [51, 52]. If a single-sport/specialization pathway is nevertheless pursued, the potential negative consequences must be carefully weighed and mitigated. Strength and injury prevention training, as well as the management of training and competition loads according to athletes’ maturation levels, are essential for reducing the risk of overuse injuries, which are common among youth handball players and are linked to sport specialization [1, 27, 32, 53–57].

The second aim was (2) to evaluate the association between multisport engagement in general and the achieved selection tier and to explore whether the breadth, depth, and type of other sports practiced within multisport players were related to selection tier. As hypothesized, being a former or current multisport player was not significantly related with selection tier in both sexes. The absence of a positive association between multisport engagement and selection tier could indicate that the benefits of multisport engagement in youth are initially latent and may only bear fruit later in adulthood [3].

To obtain a more nuanced understanding of the association between multisport engagement and selection tier, the present study explored different facets (breadth, depth, and type of other sports) of multisport engagement. In both sexes, the number of other sports practiced and the accumulated hours of training in other sports did not differentiate between selection tier, which is in line with former studies, albeit conducted in soccer [39, 40]. On the other hand, Barth et al. [21] and Güllich et al. [22] found contrasting results in their meta-analyses across various sports. They concluded that junior-age success is facilitated by intensive specialized coach-led practice in the main sport, with little or no practice in other sports. However, comparisons of studies' results across different sports are complicated because factors such as starting age, age of specialization, and TDS may vary between sports [13]. The sole study conducted in handball found that selected female players practiced more other sports and accumulated more hours of training in those sports than their non-selected peers [14]. However, this finding was not evident in male players. The authors explained the disparity between female and male players by the depth of competition for spots on a team which is higher for male players. The greater participant pool and greater competition in male players might have affected the quantity and quality of training necessary to progress through different stages of talent development. The alignment of the results for female players with those for male players in the current study, compared with the data from Baker et al. [14], may indicate either the presence of a cohort effect or an actual shift within the system over the past decade. The notion that practicing sports with similar characteristics to handball contributes to improved performance, thereby increasing the probability of selection, could only be confirmed in aesthetic sports among female players. Nevertheless, given the merely anecdotal evidence indicated by the Bayes factor, this finding should be interpreted with caution, and follow-up studies are required to draw well-supported conclusions.

The present findings suggest that multisport engagement is not detrimental for being selected as a “talent”, which is a strong argument for pursuing a multisport pathway, given its assumed numerous advantages [55]. Vice versa, sport specialization is not a prerequisite for attaining success at the youth level and consequently, it is not essential for selection into TDP [16]. Consequently, it is recommended that parents, coaches, and educators encourage youth to participate in a variety of sports during the early stages of athletic development. This will help them develop diverse motor skills and discover which sports they enjoy [1, 55]. However, the multisport pathway can substantially increase resource demands (i.e., time, effort, and finances) for athletes and their families [58]. This can make a low socioeconomic status a barrier to multisport engagement [59]. Therefore, the involvement in multiple sports should not only be endorsed but must also be facilitated.

The third aim was (3) to examine whether the handball training age could differentiate between selection tiers. Studies by Barth et al. [21] and Güllich et al. [22] have shown that junior-age sports success is facilitated by an early start in the main sport. This finding is consistent with the present results, which demonstrated a significant association between handball training age and selection tier, corroborating our initial assumptions. The difference in handball training age between NSP and YNTP players—0.87 years in females and 0.73 years in males—could be considered relevant, particularly given that it corresponds to approximately 8–11% of their total training age. The initiation of handball training at an early age allows for reaching a high handball training age in teenage years, thereby facilitating the attainment of high performance at the junior level and increasing the chance for admission to TDP and nomination for youth national teams. This may provide players with the opportunity to gain early (international) competition experience, which, in turn, has been suggested to be positively associated with long-term performance development [19, 36].

Nevertheless, youth players with a high training age are equipped with an advantage in talent selection when compared to their less experienced peers, resulting in a selection bias that favors players with a higher training age. This is problematic since the commencement of handball training at an early age cannot simply be equated with “talent”. Its initial advantage may diminish over the course of a player’s career and be less important for adult performance. This assertion is corroborated by studies which demonstrated that an early start in game sports did not serve as a meaningful predictor for future sporting success in adulthood [15, 21, 22, 25]. Consequently, training age, akin to relative age or maturation, is a characteristic that offers benefits in talent selection during youth but provides no significant advantages for sports success in later adulthood [46]. To mitigate training age selection bias, players’ performance and developmental potential should always be evaluated in consideration of their training age during selection processes. In addition, given the relatively late specialization age in handball, starting and/or specializing at an early age should not be a prerequisite for opportunities to develop talent [6, 13]. Therefore, keeping the door to TDP open for an extended duration enables talented players with lower training age to catch up on handball experience and enter TDP at a later stage [6, 60].

### Limitations

Readers should be mindful of the limitations of this study. First, the number of years spent practicing handball and other sports was used as a proxy for the training age and depth of multisport engagement, respectively. However, the years of training may not have reflected the actual number of practice hours accumulated (i.e., total training volume) during those years [15]. This would have provided more accurate metrics for deliberate practice and multisport engagement. Second, youth engagement in sport has many more nuances than a simple dichotomy of sports specialization versus multisport engagement can capture. Aspects such as playfulness (practice versus play), supervision (peer-led versus coach-led), and timing (early versus late) should also be considered [7, 8, 22, 61, 62]. In the present study, multisport engagement was limited to coach-led practice since the respective items asked for sports involvement in a club. However, it has been shown that peer-led play has negligible effects on both junior and senior performance [21, 22].

## Conclusion

The prevalence rates of sport specialization were high among female and male youth handball players at the time of the SelCamp, which was expected. Nonetheless, almost one-third of the players have never been involved in sports other than handball, indicating potential room for improvement, especially given the numerous advantages associated with multisport engagement [3, 6, 24, 28, 33]. Pursuing a multisport pathway was not a hindrance for achieving high selection tiers and thus can be considered a worthwhile alternative to the single-sport approach.

The initiation of handball training at an early age is necessary to attain a high training age by the time talent selection processes commence, which appears to be conducive to achieving high performance and for being selected as a “talent” for youth national teams or TDP. However, the initial advantages of a high training age in talent selection may become less important by adulthood [15, 21, 22, 25]. Consequently, players’ training age must be considered when evaluating performance and potential in selection processes, to mitigate possible training age selection bias.

Altogether, the findings suggest that talent research should not exclusively focus on the end result in adulthood; rather, it should also encompass the intermediate steps of talent selection processes on the road that leads toward adult high performance. This approach facilitates the acquisition of a more nuanced understanding of talent development.

## Data Availability

The data that support the findings of this study are available upon reasonable request and only with the permission of the data proprietor Deutscher Handballbund e.V. Requests to access these datasets should be directed to Prof. Dr. Dirk Büsch, dirk.buesch@uni-oldenburg.de.

## Abbreviations

ANOVA: Analysis of variance
*BF*: Bayes factor
CI: Confidence interval
DHB: Deutscher Handballbund e.V.
*LL*: Lower limit
NSP: Non-selected players
SelCamp: National Talent Selection Camp
SP: Selected player
TDP: Talent development program
TDS: Talent development system
*UL*: Upper limit
*V*: Cramér’s *V*
YNTP: Youth national team player
